# Improving subacute management of post concussion symptoms: a pilot study of the Melbourne Paediatric Concussion Scale parent report

**DOI:** 10.2217/cnc-2021-0007

**Published:** 2020-06-10

**Authors:** Gavin A Davis, Vanessa C Rausa, Franz E Babl, Katie Davies, Michael Takagi, Alison Crichton, Audrey McKinlay, Nicholas Anderson, Stephen JC Hearps, Cathriona Clarke, Remy Pugh, Kevin Dunne, Peter Barnett, Vicki Anderson

**Affiliations:** 1 Murdoch Children’s Research Institute, Melbourne, 3052, Australia; 2 Department of Neurosurgery, Austin Hospital, 3084, and Cabrini Hospital, 3144, Melbourne, Australia; 3 Department of Paediatrics, University of Melbourne, Melbourne, 3052, Australia; 4 Emergency Department, Royal Children's Hospital, Melbourne, 3052, Australia; 5 School of Psychological Sciences, University of Melbourne, Melbourne, 3052, and Turner Institute for Brain and Mental Health, Monash University, 3800, Melbourne, Australia; 6 Department of Psychology, University of Canterbury, Ilam, 8041, New Zealand; 7 Department of Rehabilitation Medicine, Royal Children’s Hospital, Melbourne, 3052, Australia; 8 Psychology Service, Royal Children’s Hospital, Melbourne, 3052, Australia

**Keywords:** assessment, concussion, pediatric, post concussion symptoms

## Abstract

**Aim:**

To pilot a modification of the Post Concussion Symptom Inventory, the Melbourne Paediatric Concussion Scale (MPCS) and examine its clinical utility.

**Materials & methods:**

A total of 40 families of concussed children, aged 8–18 years, were recruited from the emergency department. Parent responses to the MPCS in the emergency department and 2-weeks post injury determined child symptomatic status. Association between MPCS symptom endorsement and symptomatic group status was examined.

**Results:**

All additional MPCS items were endorsed by at least 25% of the parents of symptomatic children at 2 weeks. MPCS items were classified into nine symptom domains, with most falling in mood, neurological, autonomic and vestibular domains.

**Conclusion:**

The additional items and domain classifications in the MPCS have the potential to improve subacute diagnostic precision, monitoring of clinical recovery and identification of appropriate interventions post pediatric concussion.

Concussion accounts for up to 90% of pediatric head injury emergency department (ED) presentations [1]. Defined as a traumatic brain injury induced by biomechanical forces, concussion can be diagnosed where somatic, cognitive and/or emotional symptoms, physical signs (e.g., loss of consciousness, amnesia), balance impairment, cognitive impairment or sleep/wake disturbance are observed [2]. For most children, the post concussive symptoms (PCS) associated with concussion resolve spontaneously within 4-weeks post injury [2,3]. Approximately 30% of children, however, will experience persisting PCS (pPCS) beyond 4-weeks post injury, with consequences on activity participation, psychological outcomes and quality of life [4–8].

Several concussion clinical assessment tools have been developed for use among adults, with limited evaluation of developmentally appropriate pediatric specific measures [9]. Given the heterogeneity of concussion symptoms, identification remains a primary component of concussion clinical assessment and management. Indeed, in the absence of a concussion biomarker, the diagnosis of concussion in children is reliant on assessment of signs and symptoms [3]. Symptom checklists must fulfil multiple criteria, including ability to discriminate concussed from non concussed children; demonstrate symptom evolution over time; identify symptoms across multiple functional domains; identify predictors of pPCS; provide correlation with clinical recovery; and guide interventions. Two commonly used pediatric symptom scales are the Health and Behavior Inventory (HBI) [10] and the Post Concussion Symptom Inventory (PCSI) [11]. The modified HBI has been incorporated into the Child Sports Concussion Assessment Tool 5 (Child SCAT5) [12] for acute diagnosis of concussion in children. The PCSI was adapted from the Post Concussion Scale [13,14], initially developed for adult athletes. The PCSI has previously demonstrated good psychometric properties in child populations [15]. It contains parent- and age-specific child versions. Parental involvement in the assessment and management of pediatric concussion is pivotal. With substantial knowledge about a child's functioning and behavior, relevant to concussion diagnosis and recovery, parents are often utilized for gathering information regarding symptom presentation and evolution [16]. Assessment data inclusive of parent symptom report offers greater reliability in understanding the presence and functional implications of various concussion symptoms within the context of their home and school environments, that can inform diagnosis and appropriate interventions [16].

Despite the numerous requirements of concussion symptom lists, many items are generic and non specific. For example in one study, concussed youth endorsed a similar number of symptoms on a concussion symptom list to those with depression, anxiety, chronic pain or a medical diagnosis other than concussion [17]. In another study, baseline reporting of PCSI symptoms among youth athletes with no medical problems revealed endorsement of symptoms, including fatigue, drowsiness, headache, difficulty concentrating, nervousness and worry, in the absence of a concussion [18]. A further limitation is the multideterminant etiology of symptom presentations (e.g., fatigue secondary to headache, or anxiety secondary to vestibular symptoms) [19]. While the presence of any PCS, following a direct blow or impulsive force to the head, is sufficient to diagnose concussion and monitor recovery, more nuanced information is needed to guide intervention, for example, whether headache management or psycho-education for sleep problems is required. Thus, symptom domain identification (e.g., physical, cognitive, emotional, sleep) is important [20]. Differentiating symptom domains can optimize recovery by guiding individualized clinical management and intervention [20]. Lumba-Brown and colleagues examined eight commonly utilized symptom scales and found they represented only 26% of symptoms identified by expert workgroup consensus [21]. Subtypes least represented by items on symptom lists included cervical strain, ocular-motor, vestibular and anxiety/mood subtypes, with authors concluding that enhancement of current clinical symptom scales may improve assessment and intervention [21]. Emerging research of multimodal interventions to treat pPCS highlights a growing appreciation of how the emergence and maintenance of pPCS are likely the result of varying interactions between differing domain impairments [22,23].

In our prospective, longitudinal studies, we have identified clinically important symptoms in concussed children over the course of their recovery that are not currently identified within the PCSI, which are either specific to particular functional domains or affected by multiple domains [24,25]. To address this gap, we modified the PCSI symptom checklist to create the Melbourne Paediatric Concussion Scale (MPCS) with the aim of improving the tool for subacute diagnostic precision, to monitor recovery and improve its utility in guiding post-concussion management. The tool was modified for the purposes of improving sub acute clinical management and intervention over time.

The aim of this pilot study was to assess the potential utility of the parent version of the MPCS for subacute diagnostic precision, to monitor recovery and guide appropriate interventions post pediatric concussion. The frequency of symptom endorsement from ED presentation to 2-weeks post injury was examined with reference to symptomatic status at 2-weeks post injury. Symptom distribution by PCS domain was also examined.

## Materials & methods

### Design

This study is a single site, prospective, longitudinal cohort study at The Royal Children's Hospital (RCH), Melbourne, Australia. Children and their families were recruited as part of a prescreening process designed to identify eligible participants for a concussion intervention trial [26]. Recruitment occurred in The RCH ED. Measures were collected in the ED and at 2-weeks (10–17 days) post injury. Ethical approval was obtained through The RCH Human Research Ethics Committee.

### Participants

For the purposes of this sub study, participants were parents of children aged between 8 and 18 years, who had sustained a concussion, with a Glasgow Coma Scale [27] score of ≥13 at the time of hospital presentation and thereafter. Concussion was defined using the Concussion in Sport Group criteria [2]. Parents were not eligible if their child met any of the following exclusion criteria: unknown mechanism of injury, non accidental injury, head injury secondary to a faint/collapse or seizure, evidence of structural brain injury on neuroimaging, multiple trauma requiring hospitalization, cervical spine injury, pre-existing congenital neuro-ophthalmological or vestibular dysfunction, central neurological conditions, severe mental health history (e.g., current suicidal ideation, substance abuse, bipolar or psychotic disorders), a complex psychosocial history (e.g., family violence, child protection involvement), pre-existing developmental disorders for which a child is not in mainstream school and/or requires a teacher’s aide, insufficient English or inability to attend visits. All families with complete MPCS Parent Report (MPCS-P) data at two time points (in the ED and at 10–17 days post injury) at the time of data analysis were included.

### Measures

Melbourne Paediatric Concussion Scale, Parent Report (MPCS-P): includes PCSI-P items plus ten clinical questions agreed upon by clinician consensus. Lead clinical researchers from multiple disciplines including neurosurgery, emergency medicine, rehabilitation, neuropsychology, clinical psychology and physiotherapy, with concussion expertise, drew on clinical experience to generate and workshop additional items until unanimous agreement. The items were intended to survey different symptom domains to improve clinician decision regarding the ideal intervention to commence treatment, based on the pattern and severity of item endorsement. Additional items and rationale for their inclusion are outlined in Table 1. The item, *‘*appears slowed down’, was also added to the MPCS-P to align with the child version. In addition to generating additional items, clinicians allocated each MPCS symptom to one or more clinical domains that were generated by consensus of the clinician group. The original PCSI items, alongside the additional ten key clinical questions, were used as part of this study. The specific phrasing and terminology of items were subsequently revised in the final version of the MPCS (see Supplementary Table 1).

**Table 1.** Melbourne Paediatric Concussion Scale additional clinical items and rationale for inclusion.

Additional clinical item	Rationale for inclusion
Develops symptoms with exercise (e.g., headache, nausea, dizziness), travel or in crowed places	To identify possible physiological or vestibular dysfunction and guide relevant assessment/treatment.
Has difficulty shifting vision in the classroom (i.e., looking from work on desk to whiteboard)	To identify possible ocular-motor dysfunction and guide relevant assessment/treatment.
Neck pain at rest or during movement	To identify possible cervical spine dysfunction and guide relevant assessment/treatment.
Does not have enough energy to do sports or exercise or play with friends	To identify fatigue due to possible mood, autonomic or hormonal dysfunction, that may be impacting sport and social participation. This may guide intervention and recommendations (i.e., education regarding pacing)
So tired it is hard for child to pay attention (reading, doing homework, other)	To identify fatigue due to cognitive, autonomic or sleep dysfunction that may manifest as cognitive symptoms, which may be impacting school participation. This may guide school-based intervention/liaison and education to parents and school.
Difficulty falling asleep or staying asleep at night	To identify sleep difficulties and guide relevant treatment and recommendations.
Worried or anxious and/or tearful	To identify symptoms of anxiety and the potential benefit of psychological intervention.
Lost appetite	To identify possible lowered appetite due to mood or hormonal dysfunction and the potential benefit of psychological intervention.
Has difficulty keeping track of things in mind	To identify possible cognitive symptoms. This may guide school-based intervention/liaison and education to parents and school.
Slower thinking	To identify possible cognitive symptoms. This may guide school-based intervention/liaison and education to parents and school.

In keeping with recent, data-based algorithms [28], a child was considered symptomatic if their parent endorsed ≥2 symptoms on the MPCS-P, at least one point above those endorsed pre injury. This previously validated PCSI cut-off score has shown that it is able to accurately classify symptomatic children [28]. Child ratings on the MPCS data were not obtained, thus were not available for the current analysis.

### Procedure

Trained research assistants screened children presenting to the ED in real-time using The RCH Electronic Medical Record database. Consent was obtained via electronic REDCap form. Parents completed the MPCS-P in the ED and again between 10 and 17-days post injury. Demographic and injury-related information were also collected.

### Data analysis

All data were collected via REDCap [29] and analyzed using Stata 13 (Statacorp, TX, USA). Summary statistics describe the total sample, with means and standard deviations used to describe continuous data and percentages used to describe categorical data. Number and percentage of participant endorsement of MPCS items were calculated for the total sample and groups by symptomatic status, alongside mean and standard deviations of symptom severity for each item. Association between symptom endorsement and symptomatic group status was analyzed using Chi-square and 95% CI.

## Results

### Sample

**Figure 1.**
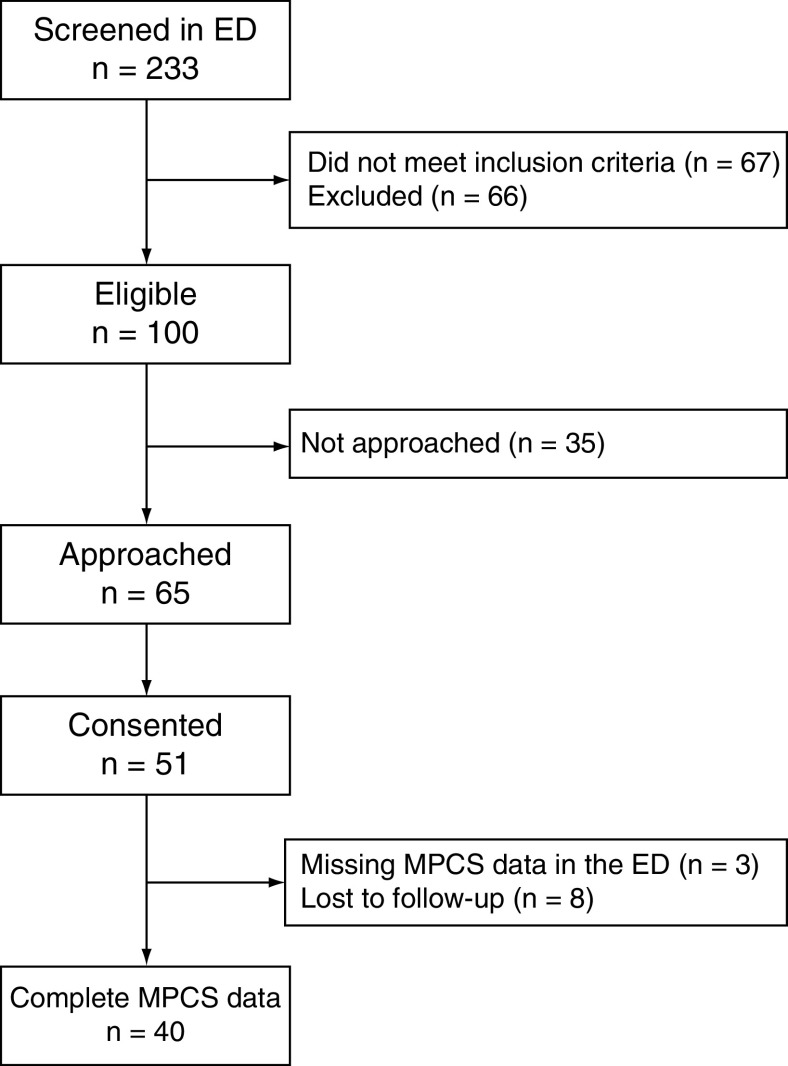
Recruitment and attrition flow chart. ED: Emergency department; MPCS: Melbourne Paediatric Concussion Scale.

Research assistants screened 233 children in the ED, of which 166 (71%) met study inclusion criteria. Of these, 66 (40%) were excluded and 35 (21%) were not approached for various reasons (e.g., missed, discharged). Of the 65 eligible families approached, 51 (78%) consented to participate in the study. There were missing MPCS-P data in the ED for three parents, and a further eight parents were lost to follow-up. Figure 1 illustrates the recruitment and attrition flow chart.

Complete MPCS-P data were available from 40 parents of concussed children. Parent and child demographics are presented in Supplementary Table 2. The mean age of concussed children was 12.7 years. Males accounted for approximately 65% of the concussed sample. The majority of children presented to the ED within 24 h post injury (90%). All children were recruited within 72 h post injury except one who presented at 8-days post injury. Eleven children (27.5%) had a history of prior concussion. At 2-weeks post injury, 23 children were symptomatic (57.5%) and 17 were asymptomatic (42.5%), based on MPCS-P report. Reference to symptomatic and asymptomatic groups hereafter refer to symptomatic status as defined at 2-weeks post injury.

### MPCS-P item endorsement at ED & 2-weeks post injury

Table 2 displays the number of parents endorsing each symptom on the MPCS in the ED and at 2-weeks, alongside measures of central tendencies for severity of item endorsed. In the ED, the most endorsed symptom on the original PCSI was ‘complains of headache’ (92.5%), followed by ‘appears or complains of dizziness’ (85.0%) and ‘appears more tired or fatigued’ (85.0%). Among the additional clinical items on the MPCS, ‘neck pain at rest or during movement’, ‘worried or anxious and/or tearful’ and ‘does not have enough energy to do sports or exercise or play with friends’ were most frequently endorsed in the ED (47.5, 45.0, 42.5% respectively).

**Table 2.** Melbourne Paediatric Concussion Scale parent item endorsement and symptom severity at emergency department and 2 weeks post-injury.

		ED (n = 40)					2 weeks (n = 40)				
		n	(%)	Mean	SD	Median	n	(%)	Mean	SD	Median
	**PCSI Item**										
1	Complains of headaches	37	(92.5)	4.0	1.4	4.0	23	(57.5)	2.5	1.5	2.0
2	Complains of nausea	29	(72.5)	4.0	1.6	4.0	12	(30.0)	2.8	1.5	3.0
3	Has balance problems	25	(62.5)	2.8	1.4	3.0	6	(15.0)	2.0	1.3	1.5
4	Appears or complains of dizziness	34	(85.0)	3.4	1.5	3.0	13	(32.5)	2.5	1.8	2.0
5	Sleeping more than usual	17	(42.5)	2.7	1.4	2.0	11	(27.5)	2.6	1.7	2.0
6	Appears drowsy	33	(82.5)	3.0	1.5	3.0	9	(22.5)	2.4	2.0	1.0
7	Sensitivity to light	15	(37.5)	2.9	1.6	2.0	9	(22.5)	1.7	1.1	1.0
8	Sensitivity to noise	15	(37.5)	2.5	1.7	2.0	10	(25.0)	2.4	1.7	1.5
9	Acts irritable	18	(45.0)	2.5	1.3	2.0	14	(35.0)	2.1	1.1	2.0
10	Appears sad	24	(60.0)	2.4	1.2	2.0	14	(35.0)	2.1	1.1	2.0
11	Acts nervous	10	(25.0)	2.0	1.1	2.0	10	(25.0)	1.9	1.0	2.0
12	Acts more emotional	22	(55.0)	2.6	1.4	2.5	17	(42.5)	2.0	1.1	2.0
13	Acts or appears mentally foggy	25	(62.5)	2.7	1.5	2.0	11	(27.5)	2.5	1.7	2.0
14	Has difficulty concentrating	22	(55.0)	2.5	1.4	2.0	15	(37.5)	2.4	1.6	2.0
15	Has difficulty remembering	18	(45.0)	2.8	1.5	3.0	12	(30.0)	2.3	1.4	2.0
16	Has or complains of visual problems (blurry, double vision)	12	(30.0)	3.3	1.4	3.0	5	(12.5)	2.2	1.8	1.0
17	Appears more tired or fatigued	34	(85.0)	3.0	1.4	3.0	15	(37.5)	2.2	1.6	2.0
18	Becomes confused with directions or tasks	14	(35.0)	2.6	1.6	2.0	10	(25.0)	2.1	1.5	1.0
19	Appears to move in a clumsy manner	14	(35.0)	2.6	1.3	3.0	6	(15.0)	2.0	1.1	2.0
20	Answers questions more slowly than usual	23	(57.5)	2.6	1.4	2.0	8	(20.0)	1.8	0.9	1.5
21	Appears slowed down	23	(57.5)	2.7	1.3	2.0	8	(20.0)	2.5	1.8	2.0
	**MPCS Item**										
1	Develops symptoms with exercise (e.g., headache, nausea, dizziness), travel or in crowded places	14	(35.0)	2.6	1.9	2.0	11	(27.5)	2.5	1.7	2.0
2	Has difficulty shifting vision in the classroom (i.e., looking from work on desk to whiteboard)	11	(27.5)	2.0	1.1	2.0	6	(15.0)	1.3	0.8	1.0
3	Neck pain at rest or during movement	19	(47.5)	2.5	1.7	2.0	7	(17.5)	2.6	2.2	1.0
4	Does not have enough energy to do sports or exercise or play with friends	17	(42.5)	2.1	1.6	1.0	8	(20.0)	2.5	1.9	2.0
5	So tired it is hard for child to pay attention (reading, doing homework, other)	15	(37.5)	1.9	1.4	1.0	8	(20.0)	2.6	2.1	2.0
6	Difficulty falling asleep or staying asleep at night	4	(10.0)	2.8	1.7	2.5	10	(25.0)	2.1	1.6	1.5
7	Worried or anxious and/or tearful	18	(45.0)	2.2	1.3	2.0	12	(30.0)	2.2	1.4	2.0
8	Lost appetite	9	(22.5)	2.2	1.1	2.0	6	(15.0)	2.2	1.3	2.0
9	Has difficulty keeping track of things in mind	14	(35.0)	1.9	1.2	1.5	8	(20.0)	2.8	1.5	2.5
10	Slower thinking	15	(37.5)	2.1	1.4	2.0	10	(25.0)	2.4	1.6	2.0

ED: Emergency department; MPCS: Melbourne Paediatric Concussion Scale; PCSI: Post-Concussion Symptom Inventory; SD: Standard Deviation.

At 2-weeks post injury, ‘complains of headache’ remained the most frequently endorsed symptom on the original PCSI (57.5%) among the entire sample, followed by ‘acts more emotional’ (42.5%), ‘appears tired or fatigued’ (37.5%) and ‘has difficulty concentrating’ (37.5%). Among the MPCS additional items, the most endorsed were ‘worried or anxious and/or tearful’ (30.0%), ‘develops symptoms with exercise (e.g., headache, nausea, dizziness), travel or in crowed places’ (27.5%), ‘difficulty falling asleep or staying asleep at night’ (25.0%) and ‘slower thinking’ (25.0%).

### Original PCSI-P endorsement among asymptomatic versus symptomatic children

Table 3 displays parent item endorsement in the ED and at 2-weeks post-injury by symptomatic status. Supplementary Tables 3 & 4 shows item endorsement by time-point and group, ranked from most frequently to least frequently endorsed by the groups. Among the original PCSI items, ‘complains of headaches’ and ‘appears or complains of dizziness’ were the most endorsed items in the ED by parents of children classified as symptomatic at 2-weeks post injury (91.3%). Headaches were the most endorsed item in the ED by parents of children later considered asymptomatic (94.1%). At 2-weeks, headaches were the most endorsed symptom by parents of both groups (symptomatic: 78.3 vs asymptomatic: 29.4%, p < 0.001).

**Table 3.** Melbourne Paediatric Concussion Scale parent item endorsement at emergency department and 2 weeks post injury by symptomatic status.

		ED							2 weeks						
		Symptomatic (n = 23)			Asymptomatic (n = 17)			Fisher’s exact test	Symptomatic (n = 23)			Asymptomatic (n = 17)			Fisher’s exact test
		n	(%)	(95% CI)	n	(%)	(95% CI)		n	(%)	(95% CI)	n	(%)	(95% CI)	
	**PCSI Item**														
1	Complains of headaches	21	(91.3)	(72.0–98.9)	16	(94.1)	(71.3–99.9)	1.000	18	(78.3)	(56.3–92.5)	5	(29.4)	(10.3–56.0)	<0.001
2	Complains of nausea	17	(73.9)	(51.6–89.8)	12	(70.6)	(44.0–89.7)	1.000	12	(52.2)	(30.6–73.2)	0	(0.0)	(0.0–19.5)	<0.001
3	Has balance problems	14	(60.9)	(38.5–80.3)	11	(64.7)	(38.3–85.8)	1.000	6	(26.1)	(10.2–48.4)	0	(0.0)	(0.0–19.5)	0.030
4	Appears or complains of dizziness	21	(91.3)	(72.0–98.9)	13	(76.5)	(50.1–93.2)	0.370	13	(56.5)	(34.5–76.8)	0	(0.0)	(0.0–19.5)	<0.001
5	Sleeping more than usual	11	(47.8)	(26.8–69.4)	6	(35.3)	(14.2–61.7)	0.530	11	(47.8)	(26.8–69.4)	0	(0.0)	(0.0–19.5)	<0.001
6	Appears drowsy	18	(78.3)	(56.3–92.5)	15	(88.2)	(63.6–98.5)	0.680	9	(39.1)	(19.7–61.5)	0	(0.0)	(0.0–19.5)	0.010
7	Sensitivity to light	9	(39.1)	(19.7–61.5)	6	(35.3)	(14.2–61.7)	1.000	9	(39.1)	(19.7–61.5)	0	(0.0)	(0.0–19.5)	0.010
8	Sensitivity to noise	8	(34.8)	(16.4–57.3)	7	(41.2)	(18.4–67.1)	0.750	8	(34.8)	(16.4–57.3)	2	(11.8)	(1.5–36.4)	0.140
9	Acts irritable	10	(43.5)	(23.2–65.5)	8	(47.1)	(23.0–72.2)	1.000	14	(60.9)	(38.5–80.3)	0	(0.0)	(0.0–19.5)	<0.001
10	Appears sad	13	(56.5)	(34.5–76.8)	11	(64.7)	(38.3–85.8)	0.750	14	(60.9)	(38.5–80.3)	0	(0.0)	(0.0–19.5)	<0.001
11	Acts nervous	5	(21.7)	(7.5–43.7)	5	(29.4)	(10.3–56.0)	0.720	10	(43.5)	(23.2–65.5)	0	(0.0)	(0.0–19.5)	<0.001
12	Acts more emotional	9	(39.1)	(19.7–61.5)	13	(76.5)	(50.1–93.2)	0.030	14	(60.9)	(38.5–80.3)	3	(17.7)	(3.8–43.4)	0.010
13	Acts or appears mentally foggy	12	(52.2)	(30.6–73.2)	13	(76.5)	(50.1–93.2)	0.190	11	(47.8)	(26.8–69.4)	0	(0.0)	(0.0–19.5)	<0.001
14	Has difficulty concentrating	12	(52.2)	(30.6–73.2)	10	(58.8)	(32.9–81.6)	0.750	13	(56.5)	(34.5–76.8)	2	(11.8)	(1.5–36.4)	0.010
15	Has difficulty remembering	9	(39.1)	(19.7–61.5)	9	(52.9)	(27.8–77.0)	0.520	12	(52.2)	(30.6–73.2)	0	(0.0)	(0.0–19.5)	<0.001
16	Has or complains of visual problems (blurry, double vision)	6	(26.1)	(10.2–48.4)	6	(35.3)	(14.2–61.7)	0.730	5	(21.7)	(7.5–43.7)	0	(0.0)	(0.0–19.5)	0.060
17	Appears more tired or fatigued	20	(87.0)	(66.4–97.2)	14	(82.4)	(56.6–96.2)	1.000	15	(65.2)	(42.7–83.6)	0	(0.0)	(0.0–19.5)	<0.001
18	Becomes confused with directions or tasks	7	(30.4)	(13.2–52.9)	7	(41.2)	(18.4–67.1)	0.520	10	(43.5)	(23.2–65.5)	0	(0.0)	(0.0–19.5)	<0.001
19	Appears to move in a clumsy manner	7	(30.4)	(13.2–52.9)	7	(41.2)	(18.4–67.1)	0.520	6	(26.1)	(10.2–48.4)	0	(0.0)	(0.0–19.5)	0.030
20	Answers questions more slowly than usual	13	(56.5)	(34.5–76.8)	10	(58.8)	(32.9–81.6)	1.000	8	(34.8)	(16.4–57.3)	0	(0.0)	(0.0–19.5)	0.010
21	Appears slowed down	11	(47.8)	(26.8–69.4)	12	(70.6)	(44.0–89.7)	0.200	8	(34.8)	(16.4–57.3)	0	(0.0)	(0.0–19.5)	0.010
	**MPCS Item**														
1	Develops symptoms with exercise (e.g., headache, nausea, dizziness), travel or in crowed places	9	(39.1)	(19.7–61.5)	5	(29.4)	(10.3–56.0)	0.740	11	(47.8)	(26.8–69.4)	0	(0.0)	(0.0–19.5)	<0.001
2	Has difficulty shifting vision in the classroom (i.e., looking from work on desk to whiteboard)	6	(26.1)	(10.2–48.4)	5	(29.4)	(10.3–56.0)	1.000	6	(26.1)	(10.2–48.4)	0	(0.0)	(0.0–19.5)	0.030
3	Neck pain at rest or during movement	12	(52.2)	(30.6–73.2)	7	(41.2)	(18.4–67.1)	0.540	7	(30.4)	(13.2–52.9)	0	(0.0)	(0.0–19.5)	0.010
4	Does not have enough energy to do sports or exercise or play with friends	12	(52.2)	(30.6–73.2)	5	(29.4)	(10.3–56.0)	0.200	8	(34.8)	(16.4–57.3)	0	(0.0)	(0.0–19.5)	0.010
5	So *tired* it is hard for child to pay attention (reading, doing homework, other)	8	(34.8)	(16.4–57.3)	7	(41.2)	(18.4–67.1)	0.750	8	(34.8)	(16.4–57.3)	0	(0.0)	(0.0–19.5)	0.010
6	Difficulty falling asleep or staying asleep at night	3	(13.0)	(2.8–33.6)	1	(5.9)	(0.2–28.7)	0.620	7	(30.4)	(13.2–52.9)	3	(17.7)	(3.8–43.4)	0.470
7	Worried or anxious and/or tearful	9	(39.1)	(19.7–61.5)	9	(52.9)	(27.8–77.0)	0.520	11	(47.8)	(26.8–69.4)	1	(5.9)	(0.2–28.7)	0.010
8	Lost appetite	8	(34.8)	(16.4–57.3)	1	(5.9)	(0.2–28.7)	0.050	6	(26.1)	(10.2–48.4)	0	(0.0)	(0.0–19.5)	0.030
9	Has difficulty keeping track of things in mind	8	(34.8)	(16.4–57.3)	6	(35.3)	(14.2–61.7)	1.000	8	(34.8)	(16.4–57.3)	0	(0.0)	(0.0–19.5)	0.010
10	Slower thinking	6	(26.1)	(10.2–48.4)	9	(52.9)	(27.8–77.0)	0.110	10	(43.5)	(23.2–65.5)	0	(0.0)	(0.0–19.5)	<0.001

ED: Emergency department; MPCS: Melbourne Paediatric Concussion Scale; PCSI: Post-Concussion Symptom Inventory.

**Table 4.** Melbourne Paediatric Concussion Scale symptoms categorized into domains.

	Neurological	Cognitive	Mood	Behavior	Autonomic	Sleep	Cervical	Vestibular	Hormonal
Complains of headaches	*						*		
Appears or complains of dizziness	*						*	*	
Appears more tired or fatigued					*	*			
Appears drowsy	*				*	*			
Complains of nausea	*							*	
Has balance problems	*						*	*	
Appears sad			*	*					
Answers questions more slowly than usual		*							
Acts or appears mentally foggy	*	*	*	*					
Has difficulty concentrating		*	*						
Neck pain at rest or during movement							*		
Doesn't have enough energy to do sports or exercise or play with friends			*		*				*
Sleeping more than usual			*			*			
Appears slowed down		*	*	*					
Acts irritable			*	*					
Sensitivity to light									
Acts more emotional	*		*						
Has difficulty remembering		*							
Develops symptoms with exercise (e.g., headache, nausea, dizziness), travel or in crowded places	*				*			*	
Worried or anxious and/or tearful			*						
Sensitivity to noise	*								
So *tired* it is hard for child to pay attention (reading, doing homework, other)		*			*	*			
Lost appetite			*						*
Has difficulty keeping track of things in mind		*							
Becomes confused with directions or tasks		*							
Appears to move in a clumsy manner	*							*	
Has or complains of visual problems (blurry, double vision)	*							*	
Has difficulty shifting vision in the classroom (i.e., looking from work on desk to whiteboard)	*							*	
Slower thinking		*							
Acts nervous			*	*					
Difficulty falling asleep or staying asleep at night						*			

In the ED, all items that were endorsed by more than 35% of parents of children who went onto be symptomatic at 2-weeks were also endorsed by more than 35% of parents of children who were recovered by 2-weeks post injury. There were no statistically significant differences on item endorsement in the ED between groups later classified as symptomatic or asymptomatic, with the exception of ‘acts more emotional’, which was less endorsed by parents of children who went onto be symptomatic relative to parents of children were recovered by 2-weeks (39.1 vs 76.5%, p = 0.030).

Of the ten items endorsed in the ED by more than 50% of parents of children who went onto be symptomatic at 2-weeks, six continued to be endorsed by more than 50% of parents of this group at 2-weeks. At 2-weeks, every PCSI symptom was endorsed by at least 20% of parents of symptomatic children. Symptoms across emotional and cognitive domains were more frequently endorsed by parents of the symptomatic group at 2-weeks relative to symptom endorsement in the ED. The items ‘acts irritable’, ‘appears sad’, ‘acts nervous’, ‘acts more emotional’, ‘difficulty concentrating’, ‘difficulty remembering’ and ‘becomes confused with directions or tasks’ were more commonly endorsed at 2-weeks as compared with the ED, while the endorsement of other items decreased.

In the asymptomatic group, only four original PCSI items were endorsed by parents at 2 weeks, each by two to five participants (11.8% [95% CI: 1.5–36.4%] to 29.4% [95% CI: 10.3–56.0%]).

### MPCS-P key clinical questions endorsement among asymptomatic versus symptomatic children

Among the additional key clinical questions, ‘neck pain at rest or during movement’ (52.2%), and ‘does not have enough energy to do sports or exercise, or play with friends’ (52.2%), were the most endorsed items in the ED by parents of children who later were classified as symptomatic at 2-weeks. Among those recovered by 2-weeks, ‘worried or anxious and/or tearful’ and ‘slower thinking’ were the most frequently endorsed by parents in the ED (52.9%).

At 2-weeks, all additional items were endorsed by at least 25% of parents of the symptomatic group. The items ‘develops symptoms with exercise (e.g., headache, nausea, dizziness), travel or in crowded places’ (47.8%, p < 0.001) and ‘worried or anxious and/or tearful’ (47.8%, p = 0.010) were the most highly endorsed by parents of the symptomatic group at this time-point. The items ‘develops symptoms with exercise, travel or in crowded places’, ‘difficulty falling asleep or staying asleep at night’, ‘worried or anxious and/or tearful’ and ‘slower thinking’ were more frequently endorsed by parents of symptomatic children at 2-weeks post injury as compared with their ratings in the ED.

Among parents of children who had recovered at 2-weeks post injury, only two additional key clinical items were endorsed at the 2-week time point; ‘difficulty falling asleep or staying asleep at night’ (17.7%, p = 0.470) and ‘worried or anxious and/or tearful’ (5.9%, p = 0.010).

### Symptom domains for PCSI-P and MPCS-P

Table 4 displays the nine post concussion symptom domains generated by clinician consensus, alongside the authors' allocation of each symptom to one or more of the domains. The MPCS additional symptoms provide 100% of symptoms in the hormonal domain and 0% in the behavior domain. In other symptom domains, provision of MPCS additional symptoms are neurological 17%, cognitive 33%, mood 27%, autonomic 60%, sleep 40%, cervical 25% and vestibular 29%.

### MPCS-P domain endorsement by asymptomatic versus symptomatic children at 2-weeks post injury

Parent endorsement of MPCS items by symptom domain and symptomatic status is shown in Supplementary Table 5. The majority of original PCSI items endorsed by parents of symptomatic children fell in the neurological and mood domains (95.7%). Among parents of asymptomatic children, the majority of PCSI items endorsed fell in the neurological (35.3%), cervical (29.4%) and mood domains (23.5%).

Of the additional items, the majority of items endorsed by parents of the symptomatic group fell in the mood (60.9%), neurological, autonomic and vestibular domains (all 56.5%). Among parents of asymptomatic children, only sleep (17.6%) and mood (5.9%) domains were endorsed.

## Discussion

Utilization of the MPCS-P has the potential to improve subacute diagnostic precision of symptomatic domains, clinical monitoring of recovery and identify appropriate intervention pathways for children and adolescents with pPCS. The MPCS-P symptoms allow assessment of additional symptom domains that would otherwise not be assessed with the PCSI-P in the subacute period.

Among a sample of 3063 children and adolescents, natural PCS recovery was shown to plateau between 2 and 4 weeks post-injury [30], highlighting the 2-week time point as pivotal to identifying those at risk for pPCS and identifying appropriate pathways for intervention. While females were shown to demonstrate a slower recovery as compared with males in that large sample [30], the current pilot study did not have a sufficient sample size for the examination of sex differences.

In the absence of reliable, valid, cost effective and readily available concussion biomarkers, symptom scales continue to be the cornerstone of concussion assessment, for both diagnosis and recovery. While the PCSI was originally developed and validated for its ability to discriminate concussed from non concussed children [11], it was not developed to specifically monitor recovery in the individual child or identify appropriate treatment pathways. Early identification of potential avenues for treatment based on endorsement of symptomatic domains is critically important to assist family counselling and introducing early therapeutic interventions [26]. We have identified MPCS-P symptoms that are endorsed more frequently by parents of children with persisting symptoms at 2 weeks. The identification of these symptoms can improve clinical management and intervention, thus reducing the risk of the emergence of secondary symptoms, such as lowered mood or anxiety, which can reinforce or amplify persisting symptoms [19].

Symptom scales such as the PCSI and HBI were developed and validated utilizing traditional post-concussion domains. The PCSI used four factors: physical/somatic, sleep/fatigue, emotional and cognitive [11], while the HBI used cognitive, somatic, emotional and behavioral [10]. Restricting symptom lists to these domains limits their utility acutely and at follow-up. This is exemplified by the requirement to modify the HBI symptom list used in the Child SCAT5 with the addition of the neck pain symptom [12]. It is evident that children with pPCS may endorse symptoms from multiple domains and that these domains may evolve over time. Further, treatment paradigms are required to address all symptomatic domains. Symptom lists that fail to identify all symptomatic domains expose the concussed child to exclusion from appropriate treatment paradigms.

Each MPCS-P symptom was assessed by consensus as belonging to one or more symptom domains. It is apparent that some symptoms are non specific, but may be endorsed within multiple domains. For example, ‘doesn't have enough energy to do sports or exercise, or play with friends’ may be due to a mood disorder, autonomic dysfunction or hormonal dysfunction. Therefore, each domain requires multiple symptoms within the checklist so that improved domain specificity may guide treatment interventions appropriately. The MPCS addresses some of the shortcomings of other symptom lists, however it is evident that the PCSI bias toward neurological, cognitive and mood domains continues to influence the bias of the MPCS-P. Further observational studies with larger sample sizes are required to investigate factor structure and the discriminant value of the additional MPCS-P symptoms in domain allocation and therapeutic management.

Validation studies for other symptom lists such as HBI [10] and PCSI [11] favored exclusion of symptoms with lower rates of endorsement from concussed children. However, those studies were designed to discriminate between concussed and non concussed children acutely and were not concerned with exploring subacute symptoms and evolution of symptoms in all nine domains over time, nor the identification of predictive symptoms of persisting symptoms at 2 weeks post injury. We have demonstrated a trend of the MPCS-P to identify symptoms that may be predictive of pPCS, and to assess these symptoms over time. The fact that some of these symptoms are endorsed less frequently than others may be a reflection of the incidence of different domain expression rather than the inadequacies of the individual symptom item.

There are a number of limitations of this study. The MPCS-P symptoms were reported by parents, which might over- or under-estimate the child’s true symptom experience depending on the degree of symptom externalization. Thus, correlation between parent and child-reporters cannot be assumed. Nevertheless, studies have demonstrated that similar information is ascertained when collecting either parent or child data alone [31]. Physiological biomarkers for each domain are not readily available, and the symptom allocation to domains is assumptive. The MPCS-P follow-up was limited to 2-weeks and prediction of persistent symptoms beyond this time requires further validation. Further longitudinal data examining the trajectories of MPCS-P symptoms will assist to establish the utility of the additional items in tracking recovery or identifying those at risk for persisting symptoms. The consensus method for allocation of symptom domains requires formal validation. Finally, previously established cut-points for determining symptomatic status based only on the original PCSI items were not re-assessed using the MPCS-P items and these cut-points require validation with the new list.

## Conclusion

The current pilot study supports the potential of the MPCS-P as a more clinically meaningful symptom checklist in the clinical assessment of children post-concussion. The additional clinical items offer a broader insight into pediatric symptom presentations, which is important for predicting risk of pPCS and determining symptom resolution over time. Further, the categorization of affected concussion domains can inform appropriate treatment, enhancing targeted intervention for recovery. Further research is required to test the predictive value of the MPCS-P beyond 2-weeks post injury.

**Summary points**

- The Melbourne Paediatric Concussion Scale addresses some of the shortcomings of other symptom lists.
- The Melbourne Paediatric Concussion Scale provides clinically meaningful information, above that of the original Post Concussion Symptom Inventory version, that can be used to guide intervention following pediatric concussion.
