# Supplementary material for: Improving subacute management of post concussion symptoms: a pilot study of the Melbourne Paediatric Concussion Scale parent report
Source: Concussion. 2020 Jun 10;7(1):CNC97. doi: 10.2217/cnc-2021-0007 (PMC9199568; doi:10.2217/cnc-2021-0007)
Supplement: Supplementary file 1 [file cnc-07-97-s1.docx]

| **Table 1. Melbourne Paediatric Concussion Scale (MPCS) Parent Report Items** | |
| --- | --- |
| Your child has or complains of: | |
| 1 | Headaches |
| 2 | Drowsiness |
| 3 | Sleeping more than usual |
| 4 | Tiredness/fatigue |
| 5 | Dizziness |
| 6 | Balance problems |
| 7 | Nausea |
| 8 | Sensitivity to light |
| 9 | Sensitivity to noise |
| 10 | Visual problems (blurry, double vision) |
| 11 | Being more clumsy |
| 12 | Being mentally “foggy” |
| 13 | Being confused with directions or tasks |
| 14 | Difficulty concentrating |
| 15 | Difficulty remembering |
| 16 | Answering questions slower than usual |
| 17 | Being more emotional |
| 18 | Being more irritable |
| 19 | Being more sad |
| 20 | Being more nervous |
| 21 | Feeling slowed down |
| 22 | Not having *enough energy* to do sports or exercise, or play with friends |
| 23 | Being so *tired* it is hard for child to pay attention (reading, doing homework, other) |
| 24 | Difficulty falling asleep or staying asleep at night |
| 25 | Developing symptoms with exercise (e.g., headache, nausea, dizziness), travel or in crowded places |
| 26 | Difficulty shifting vision in the classroom (i.e., looking from work on desk to whiteboard) |
| 27 | Neck pain at rest or during movement |
| 28 | Being worried or anxious and/or tearful |
| 29 | Loss of appetite |
| 30 | Difficulty keeping track of things in mind |
| 31 | Slower thinking |

| **Table 2. Participant demographics** |  |  |
| --- | --- | --- |
|  | Concussion (n = 40) | |
| **PARENT** |  |  |
| **Parental status** |  |  |
| Mother, n (%) | 33 | (82.50) |
| **Age group** |  |  |
| 20 - 30 years, n (%) | 1 | (2.50) |
| 31 - 40 years, n (%) | 8 | (20.00) |
| 41 - 50 years, n (%) | 22 | (55.00) |
| 50+ years, n (%) | 9 | (22.50) |
| **Main language spoken at home** |  |  |
| English, n (%) | 35 | (87.50) |
| **Marital/partner status** |  |  |
| Married or domestic partnership, n (%) | 33 | (82.50) |
| Divorced or separated, n (%) | 5 | (12.50) |
| Widowed, n (%) | 2 | (5.00) |
| **Education** |  |  |
| Less than Year 12, n (%) | 3 | (7.50) |
| Year 12 or equivalent, n (%) | 10 | (25.00) |
| University degree, n (%) | 27 | (67.50) |
| **Employment status** |  |  |
| Full time, n (%) | 21 | (52.50) |
| Part time, n (%) | 12 | (30.00) |
| Unemployed, n (%) | 2 | (5.00) |
| Not in labour force, n (%) | 5 | (12.50) |
| **CHILD** |  |  |
| **Age [years]** |  |  |
| Mean (SD) | 12.66 | (2.54) |
| Median (IQR) | 13.19 | (10.20-14.69) |
| **Time since injury to ED presentation** |  |  |
| Day of injury, n(%) | 36 | (90.00) |
| Next day to 72 hours post-injury, n(%) | 3 | (7.50) |
| >72 hours post-injury, n(%) | 1 | (2.50) |
| **Gender** |  |  |
| Male, n (%) | 26 | (65.00) |
| **Previous Concussions** |  |  |
| Any previous concussions, n (%) | 11 | (27.50) |
| Hospitalised/CT/MRI, n (%) | 2 | (18.18) |
| **Acute Injury Characteristics*** |  |  |
| Loss of consciousness, n (%) | 11 | (27.50) |
| Vomiting, n (%) | 8 | (20.00) |
| Amnesia, n (%) | 7 | (17.50) |
| Seizure, n (%) | 0 | (0.00) |
| CT Scan performed, n (%) | 4 | (10.00) |
| CT result clear, n (%) | 3 | (75.00) |
| **Past Medical History** |  |  |
| Headaches, n (%) | 4 | (10.00) |
| Migraines, n (%) | 3 | (7.50) |
| Learning disability, n (%) | 2 | (5.00) |
| ADHD, n (%) | 0 | (0.00) |
| Anxiety, n (%) | 2 | (5.00) |
| Depression, n (%) | 1 | (2.50) |
| **Medications, n (%)** |  |  |
| *None* | 37 | (92.50) |
| *Antibiotic* | 1 | (2.50) |
| *Asthma medications* | 1 | (2.50) |
| ADHD, attention-deficit/hyperactivity disorder; CT, computed tomography; IQR, interquartile range; MRI, magnetic resonance imaging | | |
| **Missing (n=4)* |  |  |

| **Table 3a. MPCS Parent Item Endorsement in the ED ranked from most to least endorsed for participants symptomatic at 2 weeks** | | | | | |
| --- | --- | --- | --- | --- | --- |
|  | ED (n=23) | | | | |
|  | N | (%) | M | SD | Med |
| Complains of headaches | 21 | (91.3) | 4.0 | 1.5 | 4.0 |
| Appears or complains of dizziness | 21 | (91.3) | 3.5 | 1.4 | 3.0 |
| Appears more tired or fatigued | 20 | (87.0) | 2.5 | 1.1 | 2.5 |
| Appears drowsy | 18 | (78.3) | 2.9 | 1.3 | 3.0 |
| Complains of nausea | 17 | (73.9) | 4.1 | 1.6 | 4.0 |
| Has balance problems | 14 | (60.9) | 2.8 | 1.3 | 3.0 |
| Appears sad | 13 | (56.5) | 2.2 | 1.3 | 2.0 |
| Answers questions more slowly than usual | 13 | (56.5) | 2.4 | 1.5 | 2.0 |
| Acts or appears mentally foggy | 12 | (52.2) | 2.3 | 1.2 | 2.0 |
| Has difficulty concentrating | 12 | (52.2) | 1.8 | 0.7 | 2.0 |
| Neck pain at rest or during movement | 12 | (52.2) | 2.8 | 2.0 | 2.0 |
| Doesn’t have enough energy to do sports or exercise, or play with friends | 12 | (52.2) | 1.6 | 1.0 | 1.0 |
| Sleeping more than usual | 11 | (47.8) | 2.2 | 1.0 | 2.0 |
| Appears slowed down | 11 | (47.8) | 2.6 | 1.3 | 2.0 |
| Acts irritable | 10 | (43.5) | 2.2 | 1.2 | 2.0 |
| Sensitivity to light | 9 | (39.1) | 2.6 | 1.5 | 2.0 |
| Acts more emotional | 9 | (39.1) | 2.6 | 1.6 | 2.0 |
| Has difficulty remembering | 9 | (39.1) | 2.8 | 1.8 | 3.0 |
| Develops symptoms with exercise (e.g., headache, nausea, dizziness), travel or in crowed places | 9 | (39.1) | 2.9 | 2.0 | 2.0 |
| Worried or anxious and/or tearful | 9 | (39.1) | 2.1 | 1.4 | 2.0 |
| Sensitivity to noise | 8 | (34.8) | 2.4 | 1.6 | 2.0 |
| So *tired* it is hard for child to pay attention (reading, doing homework, other) | 8 | (34.8) | 1.6 | 1.4 | 1.0 |
| Lost appetite | 8 | (34.8) | 2.3 | 1.2 | 2.5 |
| Has difficulty keeping track of things in mind | 8 | (34.8) | 1.9 | 1.4 | 1.5 |
| Becomes confused with directions or tasks | 7 | (30.4) | 2.3 | 1.4 | 2.0 |
| Appears to move in a clumsy manner | 7 | (30.4) | 2.1 | 1.2 | 2.0 |
| Has or complains of visual problems (blurry, double vision) | 6 | (26.1) | 2.3 | 1.0 | 3.0 |
| Has difficulty shifting vision in the classroom (i.e., looking from work on desk to whiteboard) | 6 | (26.1) | 1.7 | 0.8 | 1.5 |
| Slower thinking | 6 | (26.1) | 2.3 | 1.4 | 2.0 |
| Acts nervous | 5 | (21.7) | 1.8 | 0.8 | 2.0 |
| Difficulty falling asleep or staying asleep at night | 3 | (13.0) | 2.7 | 2.1 | 2.0 |
| **highlighted items represent 10 added key clinical questions |  |  |  |  |  |

| **Table 3b. MPCS Parent Item Endorsement at 2 weeks post-injury ranked from most to least endorsed for participants symptomatic at 2 weeks** | | | | | |
| --- | --- | --- | --- | --- | --- |
|  | 2 weeks (n=23) | | | | |
|  | N | (%) | M | SD | Med |
| Complains of headaches | 18 | (78.3) | 2.9 | 1.5 | 3.0 |
| Appears more tired or fatigued | 15 | (65.2) | 2.2 | 1.6 | 2.0 |
| Acts irritable | 14 | (60.9) | 2.1 | 1.1 | 2.0 |
| Appears sad | 14 | (60.9) | 2.1 | 1.1 | 2.0 |
| Acts more emotional | 14 | (60.9) | 2.1 | 1.2 | 2.0 |
| Appears or complains of dizziness | 13 | (56.5) | 2.5 | 1.8 | 2.0 |
| Has difficulty concentrating | 13 | (56.5) | 2.6 | 1.6 | 2.0 |
| Complains of nausea | 12 | (52.2) | 2.8 | 1.5 | 3.0 |
| Has difficulty remembering | 12 | (52.2) | 2.3 | 1.4 | 2.0 |
| Sleeping more than usual | 11 | (47.8) | 2.6 | 1.7 | 2.0 |
| Acts or appears mentally foggy | 11 | (47.8) | 2.5 | 1.7 | 2.0 |
| Develops symptoms with exercise (e.g., headache, nausea, dizziness), travel or in crowed places | 11 | (47.8) | 2.5 | 1.7 | 2.0 |
| Worried or anxious and/or tearful | 11 | (47.8) | 2.2 | 1.5 | 2.0 |
| Acts nervous | 10 | (43.5) | 1.9 | 1.0 | 2.0 |
| Becomes confused with directions or tasks | 10 | (43.5) | 2.1 | 1.5 | 1.0 |
| Slower thinking | 10 | (43.5) | 2.4 | 1.6 | 2.0 |
| Appears drowsy | 9 | (39.1) | 2.4 | 2.0 | 1.0 |
| Sensitivity to light | 9 | (39.1) | 1.7 | 1.1 | 1.0 |
| Sensitivity to noise | 8 | (34.8) | 2.3 | 1.8 | 1.0 |
| Answers questions more slowly than usual | 8 | (34.8) | 1.8 | 0.9 | 1.5 |
| Appears slowed down | 8 | (34.8) | 2.5 | 1.8 | 2.0 |
| Doesn’t have enough energy to do sports or exercise, or play with friends | 8 | (34.8) | 2.5 | 1.9 | 2.0 |
| So *tired* it is hard for child to pay attention (reading, doing homework, other) | 8 | (34.8) | 2.6 | 2.1 | 2.0 |
| Has difficulty keeping track of things in mind | 8 | (34.8) | 2.8 | 1.5 | 2.5 |
| Neck pain at rest or during movement | 7 | (30.4) | 2.6 | 2.2 | 1.0 |
| Difficulty falling asleep or staying asleep at night | 7 | (30.4) | 2.3 | 1.9 | 1.0 |
| Has balance problems | 6 | (26.1) | 2.0 | 1.3 | 1.5 |
| Appears to move in a clumsy manner | 6 | (26.1) | 2.0 | 1.1 | 2.0 |
| Has difficulty shifting vision in the classroom (i.e., looking from work on desk to whiteboard) | 6 | (26.1) | 1.3 | 0.8 | 1.0 |
| Lost appetite | 6 | (26.1) | 2.2 | 1.3 | 2.0 |
| Has or complains of visual problems (blurry, double vision) | 5 | (21.7) | 2.2 | 1.8 | 1.0 |

| **Table 4a. MPCS Parent Item Endorsement in the ED ranked from most to least endorsed for participants asymptomatic at 2 weeks** | | | | | |
| --- | --- | --- | --- | --- | --- |
|  | ED (n=17) | | | | |
|  | N | (%) | M | SD | Med |
| Complains of headaches | 16 | (94.1) | 4.1 | 1.4 | 4.0 |
| Appears drowsy | 15 | (88.2) | 3.1 | 1.7 | 3.0 |
| Appears more tired or fatigued | 14 | (82.4) | 3.6 | 1.6 | 3.5 |
| Appears or complains of dizziness | 13 | (76.5) | 3.2 | 1.6 | 3.0 |
| Acts more emotional | 13 | (76.5) | 2.7 | 1.3 | 3.0 |
| Acts or appears mentally foggy | 13 | (76.5) | 3.1 | 1.8 | 3.0 |
| Complains of nausea | 12 | (70.6) | 4.0 | 1.5 | 4.5 |
| Appears slowed down | 12 | (70.6) | 2.8 | 1.3 | 2.5 |
| Has balance problems | 11 | (64.7) | 2.8 | 1.7 | 3.0 |
| Appears sad | 11 | (64.7) | 2.6 | 1.1 | 2.0 |
| Has difficulty concentrating | 10 | (58.8) | 3.2 | 1.6 | 3.0 |
| Answers questions more slowly than usual | 10 | (58.8) | 2.8 | 1.3 | 3.0 |
| Has difficulty remembering | 9 | (52.9) | 2.9 | 1.4 | 3.0 |
| Worried or anxious and/or tearful | 9 | (52.9) | 2.2 | 1.3 | 2.0 |
| Slower thinking | 9 | (52.9) | 2.0 | 1.4 | 1.0 |
| Acts irritable | 8 | (47.1) | 2.9 | 1.3 | 3.0 |
| Sensitivity to noise | 7 | (41.2) | 2.7 | 2.0 | 2.0 |
| Becomes confused with directions or tasks | 7 | (41.2) | 3.0 | 1.7 | 3.0 |
| Appears to move in a clumsy manner | 7 | (41.2) | 3.1 | 1.2 | 3.0 |
| Neck pain at rest or during movement | 7 | (41.2) | 1.9 | 1.1 | 2.0 |
| So *tired* it is hard for child to pay attention (reading, doing homework, other) | 7 | (41.2) | 2.3 | 1.5 | 2.0 |
| Sleeping more than usual | 6 | (35.3) | 3.5 | 1.8 | 4.0 |
| Sensitivity to light | 6 | (35.3) | 3.3 | 1.8 | 2.5 |
| Has or complains of visual problems (blurry, double vision) | 6 | (35.3) | 4.2 | 1.2 | 4.0 |
| Has difficulty keeping track of things in mind | 6 | (35.3) | 1.8 | 1.0 | 1.5 |
| Acts nervous | 5 | (29.4) | 2.2 | 1.3 | 2.0 |
| Develops symptoms with exercise (e.g., headache, nausea, dizziness), travel or in crowed places | 5 | (29.4) | 2.0 | 1.7 | 1.0 |
| Has difficulty shifting vision in the classroom (i.e., looking from work on desk to whiteboard) | 5 | (29.4) | 2.4 | 1.3 | 3.0 |
| Doesn’t have enough energy to do sports or exercise, or play with friends | 5 | (29.4) | 3.2 | 2.2 | 2.0 |
| Difficulty falling asleep or staying asleep at night | 1 | (5.9) | 3.0 | . | 3.0 |
| Lost appetite | 1 | (5.9) | 2.0 | . | 2.0 |
| **highlighted items represent 10 added key clinical questions |  |  |  |  |  |

| **Table 4b. MPCS Parent Item Endorsement at 2 weeks post-injury ranked from most to least endorsed for participants asymptomatic at 2 weeks** | | | | | |
| --- | --- | --- | --- | --- | --- |
|  | 2 weeks (n=17) | | | | |
|  | N | (%) | M | SD | Med |
| Complains of headaches | 5 | (29.4) | 0.4 | 0.6 | 0.0 |
| Acts more emotional | 3 | (17.7) | 1.7 | 0.6 | 2.0 |
| Difficulty falling asleep or staying asleep at night | 3 | (17.7) | 1.7 | 0.6 | 2.0 |
| Sensitivity to noise | 2 | (11.8) | 3.0 | 1.4 | 3.0 |
| Has difficulty concentrating | 2 | (11.8) | 1.0 | 0.0 | 1.0 |
| Worried or anxious and/or tearful | 1 | (5.9) | 2.0 | . | 2.0 |
| Complains of nausea | 0 | (0.0) | . | . | . |
| Has balance problems | 0 | (0.0) | . | . | . |
| Appears or complains of dizziness | 0 | (0.0) | . | . | . |
| Sleeping more than usual | 0 | (0.0) | . | . | . |
| Appears drowsy | 0 | (0.0) | . | . | . |
| Sensitivity to light | 0 | (0.0) | . | . | . |
| Acts irritable | 0 | (0.0) | . | . | . |
| Appears sad | 0 | (0.0) | . | . | . |
| Acts nervous | 0 | (0.0) | . | . | . |
| Acts or appears mentally foggy | 0 | (0.0) | . | . | . |
| Has difficulty remembering | 0 | (0.0) | . | . | . |
| Has or complains of visual problems (blurry, double vision) | 0 | (0.0) | . | . | . |
| Appears more tired or fatigued | 0 | (0.0) | . | . | . |
| Becomes confused with directions or tasks | 0 | (0.0) | . | . | . |
| Appears to move in a clumsy manner | 0 | (0.0) | . | . | . |
| Answers questions more slowly than usual | 0 | (0.0) | . | . | . |
| Appears slowed down | 0 | (0.0) | . | . | . |
| Develops symptoms with exercise (e.g., headache, nausea, dizziness), travel or in crowed places | 0 | (0.0) | . | . | . |
| Has difficulty shifting vision in the classroom (i.e., looking from work on desk to whiteboard) | 0 | (0.0) | . | . | . |
| Neck pain at rest or during movement | 0 | (0.0) | . | . | . |
| Doesn’t have enough energy to do sports or exercise, or play with friends | 0 | (0.0) | . | . | . |
| So *tired* it is hard for child to pay attention (reading, doing homework, other) | 0 | (0.0) | . | . | . |
| Lost appetite | 0 | (0.0) | . | . | . |
| Has difficulty keeping track of things in mind | 0 | (0.0) | . | . | . |
| Slower thinking | 0 | (0.0) | . | . | . |

| **Table 5. Parent endorsement of MPCS items by symptom domain and symptomatic status** | | | | | | | | |  | |  | |  | |  | |  | |  | |  | |
| --- | --- | --- | --- | --- | --- | --- | --- | --- | --- | --- | --- | --- | --- | --- | --- | --- | --- | --- | --- | --- | --- | --- |
|  |  |  | PCSI Symptoms | | | | MPCS Symptoms | | | | | | | MPCS Total | | | | | | | |  |
|  |  |  | Asymptomatic | | Symptomatic | | Asymptomatic | | | Symptomatic | | | | Asymptomatic | | | | Symptomatic | | | |  |
|  |  |  | n | % | n | % | n | % | | n | | % | | n | | % | | n | | % | |  |
| N | Asymptomatic | 17 |  |  |  |  |  |  | |  | |  | |  | |  | |  | |  | |  |
|  | Symptomatic | 23 |  |  |  |  |  |  | |  | |  | |  | |  | |  | |  | |  |
|  |  |  |  |  |  |  |  |  | |  | |  | |  | |  | |  | |  | |  |
| Neurological | ED |  | 17 | (100.0) | 22 | (95.7) | 9 | (52.9) | | 10 | | (43.5) | | 17 | | (100.0) | | 22 | | (95.7) | |  |
|  | 2 weeks |  | 6 | (35.3) | 22 | (95.7) | 0 | (0.0) | | 13 | | (56.5) | | 6 | | (35.3) | | 22 | | (95.7) | |  |
|  |  |  |  |  |  |  |  |  | |  | |  | |  | |  | |  | |  | |  |
| Cognitive | ED |  | 15 | (88.2) | 19 | (82.6) | 11 | (64.7) | | 14 | | (60.9) | | 15 | | (88.2) | | 21 | | (91.3) | |  |
|  | 2 weeks |  | 2 | (11.8) | 14 | (60.9) | 0 | (0.0) | | 12 | | (52.2) | | 2 | | (11.8) | | 14 | | (60.9) | |  |
|  |  |  |  |  |  |  |  |  | |  | |  | |  | |  | |  | |  | |  |
| Mood | ED |  | 16 | (94.1) | 21 | (91.3) | 10 | (58.8) | | 17 | | (73.9) | | 16 | | (94.1) | | 22 | | (95.7) | |  |
|  | 2 weeks |  | 4 | (23.5) | 22 | (95.7) | 1 | (5.9) | | 14 | | (60.9) | | 4 | | (23.5) | | 22 | | (95.7) | |  |
|  |  |  |  |  |  |  |  |  | |  | |  | |  | |  | |  | |  | |  |
| Behaviour | ED |  | 16 | (94.1) | 21 | (91.3) |  |  | |  | |  | | 16 | | (94.1) | | 21 | | (91.3) | |  |
|  | 2 weeks |  | 0 | (0.0) | 20 | (87.0) |  |  | |  | |  | | 0 | | (0.0) | | 20 | | (87.0) | |  |
|  |  |  |  |  |  |  |  |  | |  | |  | |  | |  | |  | |  | |  |
| Autonomic | ED |  | 16 | (94.1) | 20 | (87.0) | 9 | (52.9) | | 14 | | (60.9) | | 16 | | (94.1) | | 21 | | (91.3) | |  |
|  | 2 weeks |  | 0 | (0.0) | 16 | (69.6) | 0 | (0.0) | | 13 | | (56.5) | | 0 | | (0.0) | | 17 | | (73.9) | |  |
|  |  |  |  |  |  |  |  |  | |  | |  | |  | |  | |  | |  | |  |
| Sleep | ED |  | 16 | (94.1) | 20 | (87.0) | 7 | (41.2) | | 9 | | (39.1) | | 16 | | (94.1) | | 21 | | (91.3) | |  |
|  | 2 weeks |  | 0 | (0.0) | 16 | (69.6) | 3 | (17.6) | | 12 | | (52.2) | | 3 | | (17.6) | | 17 | | (73.9) | |  |
|  |  |  |  |  |  |  |  |  | |  | |  | |  | |  | |  | |  | |  |
| Cervical | ED |  | 17 | (100.0) | 22 | (95.7) | 7 | (41.2) | | 12 | | (52.2) | | 17 | | (100.0) | | 22 | | (95.7) | |  |
|  | 2 weeks |  | 5 | (29.4) | 19 | (82.6) | 0 | (0.0) | | 7 | | (30.4) | | 5 | | (29.4) | | 20 | | (87.0) | |  |
|  |  |  |  |  |  |  |  |  | |  | |  | |  | |  | |  | |  | |  |
| Vestibular | ED |  | 15 | (88.2) | 22 | (95.7) | 9 | (52.9) | | 10 | | (43.5) | | 15 | | (88.2) | | 22 | | (95.7) | |  |
|  | 2 weeks |  | 0 | (0.0) | 19 | (82.6) | 0 | (0.0) | | 13 | | (56.5) | | 0 | | (0.0) | | 20 | | (87.0) | |  |
|  |  |  |  |  |  |  |  |  | |  | |  | |  | |  | |  | |  | |  |
| Hormonal | ED |  |  |  |  |  | 5 | (29.4) | | 15 | | (65.2) | | 5 | | (29.4) | | 15 | | (65.2) | |  |
|  | 2 weeks |  |  |  |  |  | 0 | (0.0) | | 10 | | (43.5) | | 0 | | (0.0) | | 10 | | (43.5) | |  |
|  |  |  |  |  |  |  |  |  | |  | |  | |  | |  | |  | |  | |  |
